# Bronchodilator response and lung function decline: Associations with exhaled nitric oxide with regard to sex and smoking status^☆^

**DOI:** 10.1016/j.waojou.2021.100544

**Published:** 2021-05-18

**Authors:** Elisabet Nerpin, Diogenes Seraphim Ferreira, Joost Weyler, Vivi Schlunnsen, Rain Jogi, Chantal Raherison Semjen, Thorainn Gislasson, Pascal Demoly, Joachim Heinrich, Dennis Nowak, Angelo Corsico, Simone Accordini, Alessandro Marcon, Giulia Squillacioti, Mario Olivieri, Rune Nielsen, Ane Johannessen, Francisco Gómez Real, Judith Garcia -Aymerich, Isabel Urrutia, Antonio Pereira-Vega, Jose Antonio Gullón, Anna-Carin Olin, Bertil Forsberg, Össur Ingi Emilsson, Isabelle Pin, Deborah Jarvis, Christer Janson, Andrei Malinovschi

**Affiliations:** a Department of Medical Sciences, Respiratory Medicine, Allergy and Sleep, Uppsala University, Uppsala, Sweden; b Department of Medical Sciences: Clinical Physiology, Uppsala University, Uppsala, Sweden; c Department of Medicine, Health and Social Studies, Dalarna University, Falun, Sweden; d Department of Epidemiology and Preventive Medicine, School of Public Health and Preventive Medicine, Melbourne, Australia; e Alergia e Imunologia, Complexo Hospital de Clinicas, Universidade Federal do Paraná, Curitiba, Brazil; f Epidemiology and Social Medicine, University of Antwerp, Antwerp, Belgium; g Department of Environment Occupation & Health, Danish Ramazzini Centre, Aarhus University, Aarhus, Denmark; h Lung Clinic, Tartu University Hospital, Tartu, Estonia; i U1219, Bordeaux Population Health Research Center, Bordeaux, France; j Department of Sleep, Landspitali University Hospital, Reykjavik, Iceland; k Faculty of Medicine, University of Iceland, Reykjavik, Iceland; l Département de Pneumologie et Addictologie, Centre Hospitalier Universitaire de Montpellier, Hopital Arnaud de Villeneuve, Univ Montpellier, Montpellier, France; m Institut Pierre-Louis D'épidémiologie et de Santé Publique, Équipe EPAR, Sorbonne Université, INSERM, Paris, France; n Institute and Outpatient Clinic for Occupational, Social and Environmental Medicine, University Hospital Munich, Ludwig Maximilians University Munich, Munich, Germany; o Institute of Epidemiology, Helmholtz Zentrum München - German Research Center for Environmental Health, Neuherberg, Germany; p Allergy and Lung Health Unit, Melbourne School of Population and Global Health, The University of Melbourne, Melbourne, Victoria, Australia; q Hospital of the Ludwig-Maximilian University Munich, LMU Munich, Munich, Germany; r Comprehensive Pneumology Center Munich (CPC-M), German Center for Lung Research (DZL), Munich, Germany; s Division of Respiratory Diseases, Medical Sciences and Infectious Diseases Department, IRCCS Policlinico San Matteo Foundation; t Department of Internal Medicine and Therapeutics, University of Pavia, Pavia, Italy; u Unit of Epidemiology and Medical Statistics, Department of Diagnostics and Public Health, University of Verona, Verona, Italy; v Department of Public Health and Pediatrics - University of Turin, Turin, Italy; w Unit of Occupational Medicine, Department of Diagnostics and Public Health, University of Verona, Verona, Italy; x Department of Thoracic Medicine, Haukeland University Hospital, Bergen, Norway; y Department of Clinical Science, University of Bergen, Bergen, Norway; z Department of Global Public Health and Primary Care, Centre for International Health, University of Bergen, Bergen, Norway; aa Department of Occupational Medicine, Haukeland University Hospital, Bergen, Norway; ab Department of Obstetrics and Gynecology, Haukeland University Hospital, Bergen, Norway; ac ISGlobAL, Centre for Research in Environmental Epidemiology (CREAL), Barcelona, Spain; ad Pneumology Service of Galdakao Hospital in Bizkaia, Spain; ae Pneumology and Allergy Service of the Juan Ramón Jiménez Hospital in Huelva, Spain; af Peumology. San Agustín Universitary Hospital, Avilés, Spain; ag Section of Occupational and Environmental Medicine, Sahlgrenska Academy, University of Gothenburg, Gothenburg, Sweden; ah Sustainable Health, Department of Public Health and Clinical Medicine, Umeå University, Umeå, Sweden; ai CHU Grenoble Alpes, Inserm, Institut for Advanced Biosciences, Grenoble Alpes University, Grenoble, France; aj National Heart and Lung Institute, Imperial College London, London, UK

**Keywords:** Bronchodilatation, Epidemiology, FeNO, Lung function

## Abstract

**Background:**

Fractional exhaled nitric oxide (FeNO) is a marker of type-2 inflammation used both to support diagnosis of asthma and follow up asthma patients. The associations of FeNO with lung function decline and bronchodilator (BD) response have been studied only scarcely in large populations.

**Objectives:**

To study the association between FeNO and a) retrospective lung function decline over 20 years, and b) lung function response to BD among asthmatic subjects compared with non-asthmatic subjects and with regards to current smoking and sex.

**Methods:**

Longitudinal analyses of previous lung function decline and FeNO level at follow-up and cross-sectional analyses of BD response and FeNO levels in 4257 participants (651 asthmatics) from the European Community Respiratory Health Survey.

**Results:**

Among asthmatic subjects, higher percentage declines of FEV_1_ and FEV_1_/FVC were associated with higher FeNO levels (p = 0.001 for both) at follow-up. These correlations were found mainly among non-smoking individuals (p = 0.001) and females (p = 0.001) in stratified analyses.

Percentage increase in FEV_1_ after BD was positively associated with FeNO levels in non-asthmatic subjects. Further, after stratified for sex and smoking separately, a positive association was seen between FEV_1_ and FeNO levels in non-smokers and women, regardless of asthma status.

**Conclusions:**

We found a relationship between elevated FeNO and larger FEV_1_ decline over 20 years among subjects with asthma who were non-smokers or women. The association between elevated FeNO levels and larger BD response was found in both non-asthmatic and asthmatic subjects, mainly in women and non-smoking subjects.

## Introduction

Fractional exhaled nitric oxide (FeNO) is based on a non-invasive method to measure nitric oxide (NO) in exhaled air and is a biomarker of type-2 inflammation,^1^ reflecting activation of IL-4/-13-driven mechanisms.^2^ Increased FeNO is related to response to inhaled corticosteroids in both subjects with asthma^3^ and subjects with nonspecific respiratory airway symptoms.^4^ FeNO has a clinical role in the diagnostic workup of asthma and the monitoring of patients with asthma.^5^

Lung function is often assessed by measuring forced expiratory volume in 1 sec (FEV_1_) and forced vital capacity (FVC). Early identification of subjects at risk of accelerated decline in lung function is important, as irreversible airflow obstruction is known to be associated with increased morbidity and mortality.^6^ Lung function decline is faster in asthmatics than in healthy subjects.^7^^,^^8^ Factors contributing to accelerated lung function decline in patients with asthma are smoking,^7^ recurrent exacerbations,^9^ and low baseline FEV_1_.^10^ Evidence suggests that airway inflammation may play an important role in the progression of lung function impairment in asthma.^11^^,^^12^ Eosinophil inflammation appears to relate to accelerated lung function decline^13^ in both asthmatic and non-asthmatic individuals. However, FeNO has been studied only scarcely in relation to lung function decline. Evidence from small, selected populations of asthmatics suggests that higher FeNO levels predict larger lung function decline.^14^, ^15^, ^16^ To our knowledge, no studies have investigated the relation between FeNO levels and lung function decline in population-based surveys.

The relation between FeNO and bronchial hyperresponsiveness to indirect stimuli, such as exercise or mannitol, is moderate.^17^^,^^18^ In lung function testing, asthma patients show a larger significant bronchodilator (BD) response in lung function to a greater extent than those without asthma,^19^ and the BD test is used in the diagnostic workup of asthma (Global Initiative for Asthma, https://ginasthma.org). An association between increased FeNO and BD response in subjects with asthma has been found in two population-based studies.^19^

FeNO values are influenced by a number of factors, for example age,^20^ sex,^20^^,^^21^ height,^20^ atopy,^20^^,^^21^ smoking,^20^^,^^22^ respiratory infections,^20^ and environmental factors.^23^ One of the most important factors in terms of magnitude is cigarette smoking, with a mean reduction of FeNO by up to 50%, depending on the extent of cigarette consumption.^24^ Sex is another important factor, with females consistently reporting about 25% lower FeNO levels than men.^20^^,^^21^ However, sex differences are seldom accounted for in studies on FeNO, and smoking status is usually only adjusted for.

Our primary aim was to study if previous lung function decline was related to FeNO in a long-term follow-up of a population-based adult cohort and whether it differed between asthmatic and non-asthmatic subjects. Our secondary aim was to study the association between lung function response to BD and FeNO with regard to presence of asthma. Lastly, we aimed to study all these associations in relation to current smoking status and sex.

## Methods

### Study sample

This is a prospective study based on the first and third surveys in the European Community Respiratory Health Survey (ECRHS I and III), performed during the periods 1990–1994 and 2010–2013, respectively, using data from 23 centres across 10 European countries and Australia.

Briefly, ECRHS is an international multicentre population-based study with the aim to determine the prevalence of and risk factors for the development of asthma, allergic disease, atopy, and rapid loss of lung function in middle-aged adults living in Europe and Australia. ECRHS I was first performed in the early 1990s, in subjects aged 20–44 years. The subjects were randomly selected to complete a short postal questionnaire about asthma symptoms and attacks in the preceding 12 months, current use of asthma medication, and presence of nasal allergies, including hay fever. Both a random sample and a symptomatic sample of responders were then invited to attend further examinations at their study centre.

In ECRHS III, subjects who participated in the clinical part of ECRHS I were sent a short postal questionnaire. Those who responded were invited to participate in lung function and blood tests and to fill out additional questionnaires. Further details about ECRHS have been published elsewhere^25^^,^^26^ and can also be found on its website: www.ecrhs.org.

**Fig. 1:**
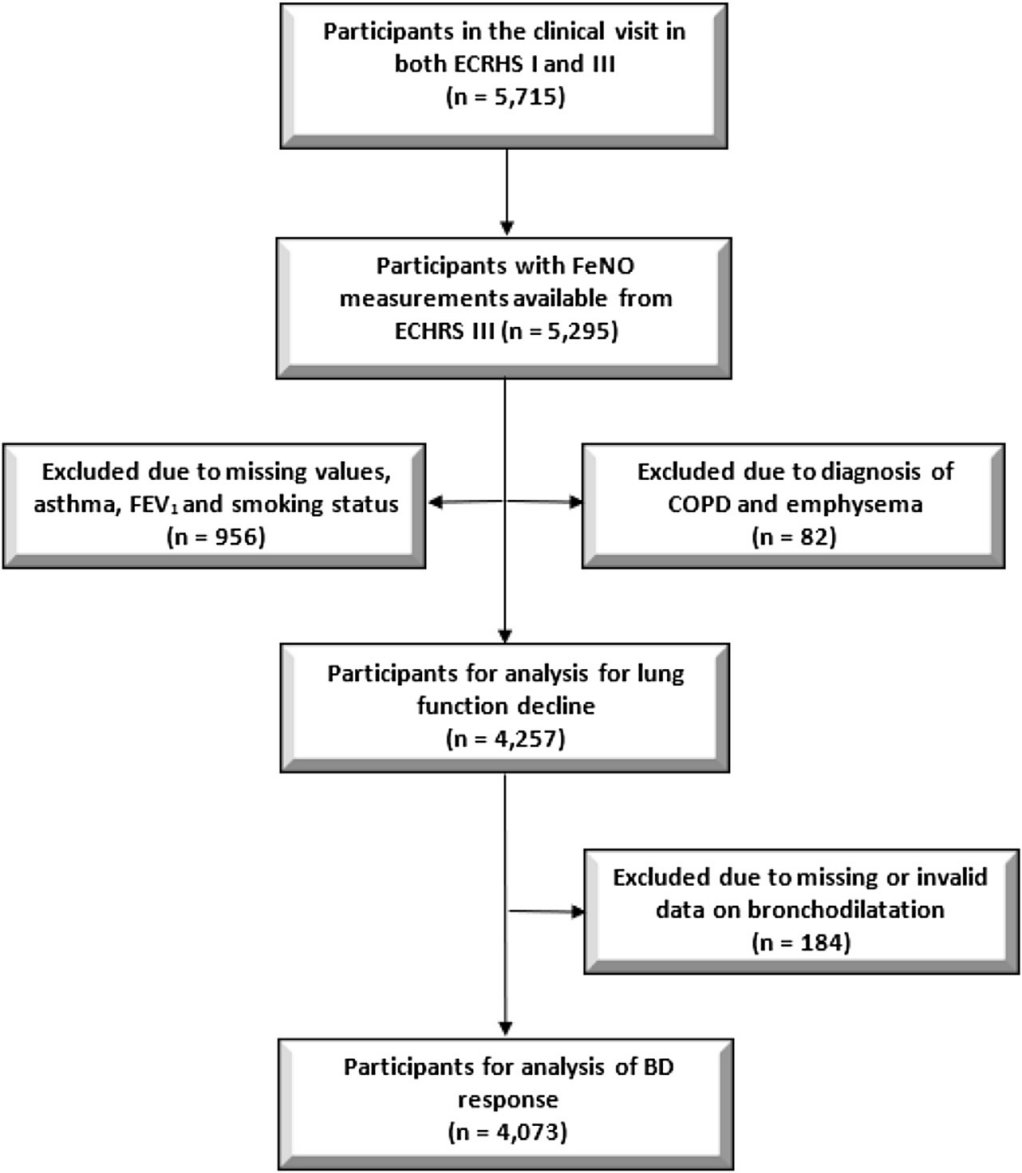
Flowchart showing the selection process and numbers of subjects available for analyses. Definitions of asthma and bronchodilator (BD) are specified in the methods section. ECRHS, European Community Respiratory Health Survey. FeNO, fractional exhaled nitric oxide

The current analyses were restricted to 5295 adults from the full cohort (random and symptomatic sample) who participated in both surveys (ECRHS I and ECRHS III) and had valid information on FeNO from ECRHS III. We excluded 956 subjects with missing values (asthma and FEV_1_) and 82 subjects with a diagnosis of chronic obstructive pulmonary disease (COPD) or emphysema, resulting in 4257 subjects. Of these, 651 had self-reported asthma. For analysis of bronchodilation, we had a total of 4073 subjects with both pre- and post-FEV_1_ data, Fig. 1.

### Questionnaires

The participants were asked in an interviewer-led questionnaire whether they had respiratory symptoms, asthma, or COPD, and about their smoking habits and use of inhaled corticosteroids in the preceding 12 months. They were also asked whether they had any nasal allergies, including hay fever.

The asthma group consisted of participants with current asthma. Current asthma was defined as self-reported asthma and reported having at least 1 asthma-related symptom (wheeze, nocturnal chest tightness or attacks of breathlessness following activity, at rest or at night) and/or reported current use of inhaled corticosteroids during the last 12 months. Asthma duration was defined as age at the clinical visit minus the self-reported age of asthma diagnosis. Chronic sinusitis with nasal polyposis were defined as self-reported doctor's diagnosis of the respective conditions.

The non-asthma group consisted of subjects that did not fulfil the criteria for current asthma.

COPD and emphysema were defined as a self-reported physician's diagnosis. Use of inhaled corticosteroids was defined in 2 ways: “ever used inhaled medicine to help breathing at any time in the last 12 months” and “used inhaled steroids (only) in the last 12 months” or “used inhaled medicine to help breathing at any time in the last 12 months” and “frequent inhaled steroid use in the last 3 months”. Oral steroid intake was defined as “used oral medicine to help breathing at any time in the last 12 months and used oral steroid tablets in the last 3 months”.

### Measurements of FeNO

FeNO measurements (only available from ECHRS III) were performed in accordance with the recommendations of the American Thoracic Society,^27^ with the exception that they were performed as single measurements^28^ by using hand-held portable sampling devices (NIOX MINO Aerocrine AB, Solna, Sweden). Patients were instructed to avoid smoking, eating, drinking, and strenuous exercise for at least 1 h before the test.

Values below 5 ppb (the lower limit of detection of the device) were recorded in 20 subjects and these individuals were given an arbitrary value of 3.5 ppb (5 divided by √2).

### Lung function

Spirometry was performed at both baseline and follow-up, but bronchodilation test was only performed in ECRHS III. Spirometry was performed using an EasyOne spirometer (ndd Medical Technologies Inc., Andover, MA, USA) in ECRHS III, but a Biomedin spirometer was used (Biomedin srl, Padua, Italy) in ECRHS I. Some centres had other equipment, but these differences are not believed to have affected the homogeneity of the results.^29^ The type of spirometer used at each centre has been presented by Accordini et al.^30^

Spirometry measurements were performed with the study person in a sitting position, wearing a nose clip, and in accordance with the instructions of the American Thoracic Society (ATS)/European Respiratory Society (ERS).^31^ All spirometry tests fulfilled the ATS data collection standards regarding validity of measurements, ie, 5 or more acceptable spirometry performances.

Variables obtained from spirometry and used in this study included FEV_1_, FVC, and FEV_1_/FVC ratio. Lung function changes (% change/year [Δ]) have been calculated with regards to ΔFEV_1_, ΔFVC, and ΔFEV_1_/FVC ratio. The calculation was performed as 100∗(value at ECRHS I - value at ECRHS III)/value at ECRHS I divided by the actual time between the baseline and follow-up, ie, a positive value represented decline.

In ECRHS III, spirometry was performed before and after 15 min after bronchodilation with 200 mcg salbutamol via a metred dose inhaler with a Volumatic spacer.

Reversibility in FEV_1_ was expressed as % change and calculated as follows: (the difference between pre- and post-bronchodilator FEV_1_ divided by pre-bronchodilator FEV_1_) ∗100.

### Anthropometry

Participants’ height and weight were measured by trained health technicians and used to calculate body mass index (BMI) (weight [kg]/height [m^2^]).

### Immunoglobulin E measurements

Allergen-specific immunoglobulin E (IgE) was measured against house dust mite, cat, and timothy grass. Analysis was performed in a single central laboratory (AMC Amsterdam) using the ImmunoCAP system (Thermo Fisher Scientific, Uppsala, Sweden). The assay was calibrated against the WHO standard for IgE with a range of 0.35–100 kU/l. A positive IgE was defined as ≥ 0.35 kU/l for one or more of house dust mite, cat, and timothy grass. In this study, data on IgE sensitisation were available only from 3542 subjects.

### Smoking

A current smoker was defined as someone who had smoked at least 20 packets of cigarettes or 360 g of tobacco in his/here life time (which equals at least 1 cigarette a day or 1 cigar a week for at least a year) and was still smoking during the month preceding the study. Non-smokers were subjects who answered questions on smoking habits and were not fulfilling the criteria for current smoker.

### Statistical methods

All analyses were performed using StataSE 15.1 (StataCorp, College Station, TX, USA). The results were summarised as arithmetic and geometric means and β coefficients with 95% confidence intervals (95% CI). Logarithmic transformation was performed for variables with right-skewed distribution (FeNO). FeNO level is described using geometric means and lung function response to BD is described using arithmetic means. Differences between groups were compared using Student's t-test.

We modelled the longitudinal impact of changes in lung function between ECRHS I to III on FeNO level (outcome) at ECRHS III. The cohort was divided into 2 groups: subjects free from asthma and subjects with asthma. Multiple linear regression analyses were stratified by sex and smoking status (2 strata: non and current smokers), adjusted for sex, age, height, atopy, inhaled steroids, smoking status, and study centre at follow-up (ECRHS III).

The interactions between sex and lung function (% change/year) between ECRHS I and ECRHS III with regards to FEV_1_, FVC, and FEV_1_/FVC ratio and, in ECHRS III, FEV_1_, FVC, and FEV_1_/FVC ratio, with BD) on FeNO were evaluated, adjusted for sex, age, height, atopy, inhaled steroids, smoking status, and study centre.

Multiple linear regression analyses were used to assess cross-sectional associations between lung function response to BD (expressed as % change) and FeNO level (outcome) in non-asthma/asthma subjects. Both groups were also stratified separately by smoking status (two strata: non and current smokers) and sex. All analyses were adjusted for sex, age, height, smoking, atopy, inhaled steroids, oral steroids, and study centre.

A p value of <0.05 was considered statistically significant.

### Ethics

Informed consent was obtained from all participants prior to inclusion in ECRHS I and III. Each study centre obtained approval for the study from their regional committee of medical research ethics in accordance with national legislation.

## Results

In ECRHS III, 2031 (47.7%) of the 4257 participants were men and 651 subjects had self-reported asthma. The mean age was 54.1 (±7.1) years. Exhaled NO levels were higher in asthmatic than non-asthmatic subjects: (geometric mean) 21.1 vs. 16.5 ppb, p < 0.001. When stratified by asthma and smoking status, non-smokers were seen to have higher FeNO levels than smokers: 23.4 vs 11.9 ppb. When stratified by sex, men were seen to have higher FeNO levels than women, regardless of asthma status, see Table 1.

**Table 1:** Baseline characteristics. All results presented as mean ± standard deviation (SD) if nothing else is stated

Variable	ECRHS III (n = 4257)	
	Non-asthma n = 3606	Asthma n = 651
Sex (men), n (%)	1775 (49.2)	256 (39.3)
Age (years)	54.2 (±7.1)	53.7 (±7.1)
FeNO (ppb)∗ (geometric mean) 95% CI	16.5 (16.2, 16.8)	21.1 (20.0, 22.3)
Men	18.4 (17.9, 18.6)	23.9 (21.9, 26.1)
Women	15.0 (14.6, 15.3)	19.6 (18.2, 21.0)
Non-Smoking	17.9 (17.6, 18.3)	23.4 (22.1, 24.8)
Smoking	11.5 (11.0, 12.0)	11.9 (10.5, 13.4)
FEV_1_ (litre)	3.1 (±0.73)	2.7 (±0.74)
FEV_1_ (% predicted)	96.2 (±13.6)	87.3 (±16.4)
ECHRS I → ECHRS III % decline/year	0.93 (±0.45)	1.00 (±0.67)
FVC (litre)	4.0 (±0.94)	3.8 (±0.93)
FVC (% predicted)	99.7 (±13.0)	95.2 (±13.7)
ECHRS I → ECHRS III % decline/year	0.57 (±0.4)	0.62 (±0.5)
FEV_1_/FVC	0.76 (±0.06)	0.73 (±0.08)
ECHRS I → ECHRS III % decline/year	0.36 (±0.27)	0.35 (±0.35)
FEV_1_ post BD (litre), n = 3440/633	3.2 (±0.73)	2.8 (±0.73)
FVC post BD (litre), n = 3406/621	4.0 (±0.94)	3.8 (±0.93)
FEV_1_/FVC-post BD %, n = 3387/621	0.79 (±0.06)	0.75 (±0.08)
Height (m)	1.70 (±0.10)	1.69 (±0.10)
Weight (kg)	78.6 (±16.4)	78.9 (±16.5)
ECHRS I → ECHRS III, kg, % decrease/year	0.43 (±0.46)	0.48 (±0.47)
BMI (kg/m^2^)	27.0 (±4.7)	27.7 (±5.5)
Smoking, n (%)		
Non-smoker	2955 (82.0)	554 (85.1)
Smoker	651 (18.0)	97 (14.9)
Inhaled corticosteroids, n (%)	24 (0.7)	128 (23.2)
Oral corticosteroids, n (%)	7 (0.2)	5 (0.9)
Atopy, n (%)	675 (21.3)	168 (44.9)
Chronic sinusitis with polyps, n (%)	34 (1.0)	18 (2.8)
Asthma duration (years)		28.4 (±15.2)

### Change in lung function over time and in relation to asthma status

Non-asthmatic subjects had lower % change per year [Δ] of FEV_1_ compared with asthmatic subjects. The same pattern was seen between non-smokers/smokers regardless of whether or not they had asthma. Men and women with asthma had higher ΔFEV_1_ than non-asthmatic subjects.

Women with non-asthma seemed to have higher ΔFEV_1_ and ΔFEV_1_/FVC ratio than men. In asthmatic subjects, women had higher % change in ΔFEV_1_/FVC ratio compared with men, see Table 2.

**Table 2:** Arithmetic means of lung function response (ECHRS I → ECHRS III, % change per year).

	ΔFEV_1_		ΔFVC		ΔFEV_1_/FVC	
	mean (95% CI)	p value	mean (95% CI)	p value	mean (95% CI)	p value
**Non-asthma**						
**ECHRS I** → **ECHRS III, % change per year**	0.93 (0.92, 0.95)		0.57 (0.56, 0.59)		0.34 (0.33, 0.34)	
**Smoking status**						
**Non-smokers (n = 2.943)**	0.91 (0.89, 0.92)		0.57 (0.55, 0.59)		0.32 (0.31, 0.33)	
**Smokers (n = 647)**	1.05 (1.01, 1.08)	<0.001	0.59 (0.56, 0.63)	0.22	0.42 (0.40, 0.44)	<0.001
**Sex**						
**Men (n = 1772)**	0.89 (0.87, 0.91)		0.56 (0.54, 0.58)		0.30 (0.28, 0.31)	
**Women (n = 1830)**	0.98 (0.96, 1.00)	<0.001	0.58 (0.56, 0.60)	0.19	0.37 (0.36, 0.39)	<0.001
**Asthma**						
**ECHRS I** → **ECHRS III, % change per year**	1.00 (0.95, 1.05)		0.62 (0.58, 0.67)		0.35 (0.33, 0.38)	
**Smoking status**						
**Non-smokers (n = 553)**	0.96 (0.67, 1.01)		0.60 (0.56, 0.65)		0.33 (0.30, 0.36)	
**Smokers (n = 97)**	1.26 (1.14, 1.38)	<0.001	0.76 (0.64, 0.87)	0.009	0.47 (0.40, 0.55)	<0.001
**Sex**						
**Men (n = 256)**	0.96 (0.88, 1.04)		0.61 (0.54, 0.68)		0.30 (0.26, 0.34)	
**Women (n = 394)**	1.03 (0.96, 1.09)	0.21	0.63 (0.58, 0.69)	0.61	0.39 (0.35, 0.42)	0.005

Abbreviations: ECRHS I = first survey of European Community Respiratory Health Survey, ECRHS III = third survey of European Community Respiratory Health Survey, Δ = % change per year, FEV_1_ = forced expiratory volume in 1 s, FVC = forced vital capacity, CI = confidence interval

### Change in lung function over time and in relation to FeNO levels

In subjects with asthma, a greater decline in ΔFEV_1_ and ΔFEV_1_/FVC ratio were associated with higher FeNO levels after adjusting for sex, age, height, weight, atopy, inhaled steroids, smoking status, and study centre, p < 0.001 for both. After stratification by smoking status, non-smoking subjects with asthma were seen to have a positive association between ΔFEV_1_, ΔFVC, and ΔFEV_1_/FVC ratio and higher FeNO levels, p < 0.001, p = 0.02, p < 0.001. Further, after stratification by sex, asthmatic women showed a positive association between ΔFEV_1_, ΔFVC and ΔFEV_1_/FVC ratio and FeNO levels, p < 0.001, p = 0.03, p = 0.001. No association was seen in non-asthmatics, see Table 3. Results were consistent after further adjustment of BMI change over 20 years of time. When oral steroids were added to the list of covariates, the results showed similar β coefficients and p values (data not shown). No interaction was seen between sex and yearly changes in lung function (FEV_1_, FVC, and FEV_1_/FVC) after adjustment for age, height, atopy, inhaled steroids, and study centre (data not shown).

**Table 3:** Multiple linear regression, lung function decline (% changes per year) and the association with FeNO in in non-asthma/asthma subjects stratified by smoking status and sex, respectively.

logFeNO	ΔFEV_1_		ΔFVC		ΔFEV_1_/FVC	
ECRHS I → ECRHS III, % change per year	β-coefficient (95% CI)	p value	β-coefficient (95% CI)	p value	β-coefficient (95% CI)	p value
**Non-asthma**						
**ECRHS I** → **ECRHS III, % change per year (All, n = 3127)**	0.003 (−0.02, 0.02)	0.76	0.0009 (−0.02, 0.02)	0.93	0.0009 (−0.03, 0.03)	0.96
**Smoking status**						
**Non-smokers (n** = **2571)**	0.01 (−0.007, 0.03)	0.18	0.001 (−0.02, 0.02)	0.91	0.02 (−0.01, 0.05)	0.27
**Smokers (n = 556)**	−0.05 (−0.09, 0.004)	0.07	−0.02 (−0.07, 0.04)	0.57	−0.05 (−0.12, 0.02)	0.18
**Sex**						
**Men (n** = **1555)**	−0.0001 (−0.03, 0.03)	0.99	0.002 (−0.03, 0.03)	0.88	−0.006 (−0.05, 0.05)	0.98
**Women (n** = **1572)**	0.005 (−0.02, 0.03)	0.73	−0.005 (−0.03, 0.02)	0.74	−0.0001 (−0.04, 0.04)	0.99
**Asthma**						
**ECRHS I-ECRHS III, % change per year (All, n = 315)**	0.13 (0.07, 0.18)	<0.001	0.07 (−0.004, 0.14)	0.06	0.23 (0.12, 0.35)	<0.001
**Smoking status**						
**Non-smokers (n = 263)**	0.16 (0.10, 0.23)	<0.001	0.10 (0.01, 0.19)	0.02	0.26 (0.13, 0.38)	<0.001
**Smokers (n = 52)**	−0.0007 (−0.18, 0.18)	0.99	−0.02 (−0.21, 0.17)	0.80	0.07 (−0.29, 0.44)	0.68
**Sex**						
**Men (n = 113)**	0.13 (−0.004, 0.27)	0.06	0.07 (−0.08, 0.21)	0.37	0.22 (0.01, 0.45)	0.06
**Women (n = 202)**	0.15 (0.08, 0.22)	<0.001	0.10 (0.008, 0.20)	0.03	0.24 (0.10, 0.38)	0.001

Abbreviations: FeNO = fractional exhaled NO nitric oxide, FEV_1_ = forced expiratory volume in 1 s, FVC = forced vital capacity, CI = confidence interval, ECRHS I = first survey of European Community Respiratory Health Survey, ECRHS III = third survey of European Community Respiratory Health Survey. Adjusted for sex, age, height, atopy, chronic sinusitis with polyps, asthma duration, inhaled corticosteroids, smoking status, and study centre

### FeNO and bronchodilator response

Asthmatic subjects had higher BD response with regards to FEV_1_ and FVC compared with non-asthmatic subjects, see Table 4.

**Table 4:** Arithmetic means of lung function response to BD (expressed as pre/post change) in non-asthma/asthma subjects stratified by smoking status and sex, respectively.

Lung function response to BD % change	FEV_1_, mean (95% CI)	p value	FVC, mean (95% CI)	p value	FEV_1_/FVC, mean (95% CI)	p value
**Non-Asthma n = 3440**	2.4 (2.2, 2.5)		−0.9 (−1.0, −0.7)		3.3 (3.2, 3.4)	
**Smoking status**						
**Non-smokers n = 2818**	2.3 (2.2, 2.5)		−1.0 (−1.1, −0.8)		3.4 (3.2, 3.5)	
**Smokers n = 622**	2.6 (2.2, 2.9)	0.27	−0.4 (−0.7, 0.3)	0.002	3.1 (2.8, 3.4)	0.06
**Sex**						
**Men (n = 1688)**	2.3 (2.1, 2.5)		−0.6 (−0.8, −0.4)		2.9 (2.7, 3.1)	
**Women (n = 1752)**	2.5 (2.3, 2.7)	0.14	−1.2 (−1.4, −0.9)	<0.001	3.7 (3.5, 3.9)	<0.001
**Asthma n = 633**	5.0 (4.4, 5.5)		1.2 (0.8, 1.7)		3.7 (3.3, 4.1)	
**Smoking status**						
**Non-smokers n = 540**	4.9 (4.3, 5.5)		1.0 (0.6, 1.5)		3.8 (3.4, 4.2)	
**Smokers n = 93**	5.5 (4.0, 7.0)	0.42	2.4 (1.2, 3.6)	0.03	3.0 (2.1, 4.0)	0.15
**Sex**						
**Men n** = **249**	4.9 (4.1, 5.8)		−1.8 (−1.1, 2.4)		3.0 (2.5, 3.6)	
**Women n** = **384**	5.0 (4.2, 5.7)	0.97	0.9 (0.3, 1.5)	0.08	4.1 (3.6, 4.6)	0.009

Abbreviations: BD = bronchodilator, FEV_1_ = forced expiratory volume in 1 s, FVC = forced vital capacity

In non-asthmatic subjects, a positive association was seen between higher % changes in FEV_1_ and FeNO levels after BD use, after adjusting for sex, age, height, weight, atopy, inhaled steroids, smoking status, and study centre, p = 0.02. No association was seen between lung function response to BD and FeNO levels in asthmatic subjects, see Table 5.

**Table 5:** Multiple linear regression analyses, lung function response to BD (expressed as % change) to FeNO in non-asthma/asthma subjects (adjusted) stratified by smoking status and sex, respectively.

logFeNO	Lung function response to BD % change FEV_1_		Lung function response to BD % change FVC		Lung function response to BD % change FEV_1_/FVC	
	β-coefficient (95% CI)	p value	β-coefficient (95% CI)	p value	β-coefficient (95% CI)	p value
**Non-asthma (n = 2993)**	0.002 (0.0003, 0.004)	0.02	0.001 (−0.0005, 0.003)	0.16	0.0007 (−0.001, 0.003)	0.49
**Smoking status**						
**Non-smokers (n = 2460)**	0.003 (0.0007, 0.004)	0.007	0.001 (−0.0008, 0.003)	0.26	0.001 (−0.0008, 0.004)	0.20
**Smokers (n = 533)**	−0.00008 (−0.004, 0.004)	0.97	0.003 (−0.002, 0.007)	0.25	−0.003 (−0.008, 0.002)	0.29
**Sex**						
**Men (n = 1486)**	0.0008 (−0.002, 0.003)	0.54	0.001 (−0.001, 0.004)	0.30	−0.0009 (−0.004, 0.002)	0.57
**Women (n = 1507)**	0.003 (0.0009, 0.006)	0.007	0.001 (−0.001, 0.003)	0.38	0.002 (−0.0003, 0.005)	0.08
**Asthma (n = 305)**	0.005 (−0.001, 0.011)	0.11	0.005 (−0.001, 0.012	0.11	0.002 (−0.007, 0.010)	0.69
**Smoking status**						
**Non-smokers (n = 250)**	0.008 (0.001, 0.015)	0.03	0.009 (0.001, 0.016)	0.02	0.002 (−0.008, 0.011)	0.73
**Smokers (n = 50)**	0.004 (−0.007 0.016)	0.46	−0.0006 (−0.015, 0.014)	0.93	0.032 (0.003, 0.06)	0.03
**Sex**						
**Men (n = 110)**	−0.002 (−0.012, 0.008)	067	0.006 (−0.006, 0.018)	0.35	−0.014 (−0.030, 0.003)	0.10
**Women (n = 195)**	0.008 (0.0006, 0.015)	0.04	0.006 (−0.002, 0.014)	0.14	0.007 (−0.003, 0.017)	0.20

Abbreviations: FeNO = fractional exhaled NO nitric oxide, BD = bronchodilator, FEV_1_ = forced expiratory volume in 1 s, FVC = forced vital capacity, CI = confidence interval. Adjusted for sex, age, height, atopy, inhaled corticosteroids, smoking status, and study centre

After stratification by smoking status, non-smoking subjects showed a positive association between higher % change in FEV_1_ and FeNO levels after BD use, whether they were non-asthmatic, p = 0.007, or asthmatic, p = 0.03. Further, when stratified by sex, women showed a positive association between higher % changes in FEV_1_ and FeNO levels after BD use, whether they were non-asthmatic, p = 0.007, or asthmatic, p = 0.04, Table 5. We also added oral steroids to our covariates; the results showed the same pattern (data not shown), except in asthmatic women where the β coefficient (95% CI) was 0.007 (−0.003, 0.02), p = 0.16. No interactions were seen between sex and BD response (FEV_1_, FVC, and FEV_1_/FVC) after adjustment for age, height, atopy, inhaled steroids, and study centre (data not shown).

## Discussion

The main finding of the present study was that a larger previous lung function decline over 20 years was associated with higher FeNO levels in asthmatics in ECRHS III. We also found that a higher BD response was associated with higher FeNO levels in non-asthmatic subjects. After subjects were stratified by sex and smoking status, the association appeared to be stronger for women and non-smokers, respectively, regardless of asthma status.

### FeNO and lung function decline over time

Eosinophil inflammation, assessed through blood cell counts, has been associated with faster decline of FEV_1_/FVC ratios and FEV_1_ in a population-based cohort of young adults.^13^ Also, Backman et al, using a similar design to that in our study, reported an association between accelerated previous decline in FEV_1_ and blood eosinophils among adults with asthma.^32^ This is in line with our study, where we found that a faster decline in lung function was associated with higher FeNO levels in asthmatics.

Asthma is frequently characterised by eosinophil inflammation, mainly due to type-2 inflammation, and FeNO levels are associated with degree of eosinophil airway inflammation.^33^ The association between FeNO and change in lung function has only been studied in selected asthma populations.^14^, ^15^, ^16^ In a prospective study by Matsunaga et al^16^ on non-smoking patients with controlled asthma (n = 140), subjects who had persistently high levels of FeNO had an accelerated decline in FEV_1_ and reduction in bronchodilator response over time. Similar results were shown by Van Veen et al*,*^14^ who demonstrated that FeNO level was associated with an excessive loss of lung function in non-smoking, difficult-to-treat asthmatics.

After stratification by smoking and sex, a positive association was seen only in non-smokers and women, respectively. The rationale for the first stratification was that current smokers have significantly lower FeNO levels,^34^^,^^35^ but faster lung function decline^7^ than non-smokers. Current smoking is a known determinant of FeNO levels in both healthy subjects^34^^,^^35^ and asthmatics.^36^ Similarly, current smoking is related to faster lung function decline both in non-asthmatic and asthmatic individuals.^37^ Therefore, current smoking is expected to relate both to lower FeNO and higher lung function decline. Moreover, as higher degree of smoking relates to further decreased FeNO^38^ and larger lung function decrease,^37^ this might explain the fact that the type-2-inflammatory signal reflected by FeNO might be impaired especially when looking at associations with lung function decline in current smokers.

With regards to sex stratification, a rationale is offered by a study by James et al demonstrating that smoking subjects with asthma had a more accelerated decline in lung function compared with non-smokers, and this decline in lung function was even greater in males with asthma.^37^ The mechanisms behind these sex differences are not fully understood, but appear to be related to differences in genetics and hormones, as well as sociocultural and environmental differences.^39^

### Bronchodilator response

Larger BD response was associated with higher FeNO levels in non-asthmatic subjects. This has not previously been studied in large populations of healthy individuals. In previous analyses, which included the populations studied herein and two other populations, we found that FeNO levels were related to the response to BD testing in asthmatics as well.^19^ In the present analysis, this was found only in non-smoking asthmatics. This may be related to power, as the previous analysis was based on a larger number of asthmatics. Other studies have also found an association between FeNO and the BD response in asthmatics.^40^ The fact that we found an association only in non-smoking asthmatics is probably related to the fact that smoking reduces FeNO levels;^35^ thus, the relation to BD response is impaired. The clinical role of FeNO in smokers with asthma is still debated.^41^

Somewhat surprisingly, we found a sex difference between FeNO and BD response, where women appeared to have a stronger response. Sex hormones has been shown to play an important role in the development and progression of asthma. In adults, experimental evidence from human cells and animal studies have shown that Th2-mediated airway inflammation is increased by oestrogen and that testosterone has anti-inflammatory effects.^42^

Previous studies have also indicated that middle-aged women are at greater risk of asthma than men.^43^^,^^44^ Possible mechanisms include smaller airways,^45^ faster lung function decline around menopause,^46^ sex differences in smoke metabolism,^47^ and immunological^48^ or hormonal differences.^49^

### Strengths and limitations

The main strength of the current study is that ECRHS is a large, multicentre, population-based cohort, which enabled us to conduct stratified analyses. It has a standardised protocol with validated questionnaires and careful attention has been paid to the quality and standardisation of lung function and exhaled NO measurements. Further, the study subjects came from different geographical areas, indicating that these findings could be valid in the general population.

Some limitations are worth mentioning. FeNO was measured only in ECRHS III, we do not have FeNO measurements at baseline or during the study period, which means that we cannot express a causal relationship. The present population was recruited from both a random sample and a symptomatic sample of responders in ECRHS. The long follow-up time and the fact that ECRHS III was the second follow-up should be taken into account. A response rate of just above 50% was found for ECRHS participants in all three surveys.^50^ Therefore, selection bias cannot be ruled out, although it is unlikely to have affected the associations found in this study.

## Conclusions

Our study shows that participants with asthma and faster lung function decline are characterised by higher levels of FeNO at follow-up. It could be speculated that achieving control of type-2 inflammation might result in slower lung function decline, but this must be tested in prospective studies. Higher FeNO levels are associated with a larger degree of response to bronchodilators. Further studies are needed to evaluate the clinical implications of our findings and to understand the sex differences observed.

## Authors’ consent for publication

All authors have read, and approved submission of the manuscript and the manuscript has not been published nor is it being considered for publication elsewhere, in whole or in part in any language.

## Author contributions

E. Nerpin, PhD and A Malinovschi PhD designed the present study analyses; analysed and interpreted the data; drafted the article and are accountable for ensuring that questions related to the accuracy or integrity of any part of the work are appropriately investigated and resolved. C Janson PhD designed ECRHS study, collected data, assisted in data interpretation, critically revised the article draft and provided intellectual content of importance to the work described. DS. Ferreira, PhD, J.Weyler PhD, V. Schlunnsen, PhD, R.Jögi PhD, C. Raherison Semjen, PhD, T. Gislason, PhD, P.Demoly, PhD, J. Heinric, PhD, D.Novac, PhD, A. Corsico, PhD, S. Accordini, PhD, A. Marcon, PhD, G. Squillacioti, PhD, M. Olivieri, PhD, R. Nielsen, PhD, A. Johannessen, PhD, F. G. Real, PhD, J. Garcia -Aymerich, PhD, I. Urrutia, PhD, A. Pereira-Veja, PhD, J. A. Gullón, PhD, A-C. Olin, PhD, B. Forsberg, PhD, Ö. I. Emilsson, PhD, I. Pin, PhD and D. Jarvis, PhD designed ECRHS study and/or collected data, contributed to data interpretation, critically revised the article draft and provided intellectual content of importance to the work described. All authors approved the manuscript.

## Availability of data and material

Due to ethical and legal restrictions in respective participating country, the data underlying this study are available upon reasonable request to qualified researchers. Requests for data access should be direct to the corresponding author and will be handled by the ECRHS Steering Committee.

## Ethics approval

Informed consent was obtained from all participants prior to inclusion in ECRHS I and III. Each study centre obtained approval for the study from their regional committee of medical research ethics in accordance with national legislation.

## Declaration of competing interest

DF reports grants from Asthma Foundation of Victoria, grants from Allen and Hanbury’s, grants from National Health & Medical Research Council, during the conduct of the study. VS reports grants from The Wood Dust Foundation (Project No. 444508795), during the conduct of the study. RJ reports grants from Estonian Research Council Personal Research Grant no 562, during the conduct of the study; personal fees from Consultancy, grants from Grants/grants pending, personal fees from Payment for lectures, personal fees from Travel/accommodations/meeting expenses, outside the submitted work. PD reports grants from ALK, Stallergenes Greer, grants from AstraZeneca, ThermoFisherScientific, Ménarini, grants from Bausch & Lomb, personal fees from Sanofi, Regeneron, outside the submitted work. RN reports grants and personal fees from AstraZeneca, grants from Novartis, grants from Boehringer Ingelheim, grants and personal fees from GlaxoSmithKline, outside the submitted work. IP reports other from NOVARTIS, other from ASTRA ZENECA, personal fees from AGIRadom, outside the submitted work. All other authors declare no conflicts of interests.

**The following grants contributed to funding ECRHS I**.

**Australia:** Asthma Foundation of Victoria, Allen and Hanbury’s. **Belgium**: Belgian Science Policy Office, National Fund for Scientific Research. **Denmark:** The Danish Lung Association. **Estonia:** Estonian Science Foundation, grant no 1088. **France:** Ministère de la Santé, Glaxo France, Institut Pneumologique d'Aquitaine, Contrat de Plan Etat-Région Languedoc-Rousillon, CNMATS, CNMRT (90MR/10, 91AF/6), Ministre delegué de la santé, RNSP, France; GSF. **Germany**: Bundesminister für Forschung und Technologie. **Greece:** The Greek Secretary General of Research and Technology, Fisons, Astra and Boehringer-Ingelheim. **India:** Bombay Hospital Trust. **Italy:** Ministero dell'Università e della Ricerca Scientifica e Tecnologica, CNR, Regione Veneto grant RSF n. 381/05.93. **New Zealand:** Asthma Foundation of New Zealand, Lotteries Grant Board, Health Research Council of New Zealand. **Norway:** Norwegian Research Council project no. 101422/310. **Portugal:** Glaxo Farmacêutica Lda, Sandoz Portugesa. **Spain:** Fondo de Investigación Sanitaria (#91/0016-060-05/E, 92/0319 and #93/0393), Hospital General de Albacete, Hospital General Juan Ramón Jiménez, Dirección Regional de Salud Pública (Consejería de Sanidad del Principado de Asturias), CIRIT (1997 SGR 00079), and Servicio Andaluz de Salud. **Sweden:** The Swedish Medical Research Council, the Swedish Heart Lung Foundation, and the Swedish Association against Asthma and Allergy. **Switzerland:** Swiss National Science Foundation grant 4026-28099. **UK:** National Asthma Campaign, British Lung Foundation, Department of Health, South Thames Regional Health Authority. **USA:** United States Department of Health, Education and Welfare Public Health Service (grant #2 S07 RR05521-28). The coordination of this work was supported by the European Commission.

**The following grants contributed to funding ECRHS III**.

**Australia**: National Health & Medical Research Council. **Belgium:** Antwerp South, Antwerp City – Research Foundation Flanders (FWO), grant code G.0.410.08.N.10 (both sites). **Estonia:** Tartu – SF0180060s09 from the Estonian Ministry of Education. **France:** (All) Ministère de la Santé, Programme Hospitalier de Recherche Clinique (PHRC) national 2010. Bordeaux: INSERM U897 Université Bordeaux Segalen. Grenoble: Comite Scientifique AGIRadom 2011. Paris: Agence Nationale de la Santé, Région Ile de France, domaine d’intérêt majeur (DIM). **Germany:** Erfurt: German Research Foundation HE 3294/10-1. Hamburg: German Research Foundation MA 711/6-1, NO 262/7-1. **Iceland:** Reykjavik, The Landspítali University Hospital Research Fund, University of Iceland Research Fund, ResMed Foundation, California, USA, Orkuveita Reykjavíkur (Geothermal plant), Vegagerðin (The Icelandic Road Administration (ICERA). **Italy:** All Italian centres were funded by the Italian Ministry of Health, Chiesi Farmaceutici SpA. Verona received additional funding from the Cariverona foundation, Education Ministry (MIUR). **Norway:** Norwegian Research Council grant no 214123, Western Norway Regional Health Authorities grant no 911631, Bergen Medical Research Foundation. **Spain:** Fondo de Investigación Sanitaria (PS09/02457, PS09/00716 09/01511) PS09/02185 PS09/03190), Servicio Andaluz de Salud, Sociedad Española de Neumología y Cirurgía Torácica (SEPAR 1001/2010), Fondo de Investigación Sanitaria (PS09/02457). Barcelona: Fondo de Investigación Sanitaria (FIS PS09/00716). Galdakao:Fondo de Investigación Sanitaria (FIS 09/01511). Huelva: Fondo de Investigación Sanitaria (FIS PS09/02185) and Servicio Andaluz de Salud. Oviedo: Fondo de Investigación Sanitaria(FIS PS09/03190). **Sweden:** All centres were funded by the Swedish Heart and Lung Foundation, the Swedish Asthma and Allergy Association, the Swedish Association against Lung and Heart Disease, and the Swedish Research Council for Health, Working Life and Welfare (FORTE). Göteborg also received further funding from the Swedish Council for Working Life and Social Research. Umeå also received funding from a Västerbotten County Council ALF grant. **Switzerland:** The Swiss National Science Foundation (grants no 33CSCO-134276/1, 33CSCO-108796, 3247BO-104283, 3247BO-104288, 3247BO-104284, 3247-065896, 3100-059302, 3200-052720, 3200-042532, 4026-028099), The Federal office for Forest, Environment and Landscape, The Federal Office of Public Health, The Federal Office of Roads and Transport, the canton’s government of Aargan, Basel-Stadt, Basel-Land, Geneva, Luzern, Ticino, Valais, and Zürich, the Swiss Lung League, the canton’s Lung League of Basel Stadt/ Basel, Landschaft, Geneva, Ticino, Valais, and Zurich, SUVA, Freiwillige Akademische Gesellschaft, UBS Wealth Foundation, Talecris Biotherapeutics GmbH, Abbott Diagnostics, European Commission 018996 (GABRIEL), Wellcome Trust WT 084703MA, **UK:** Medical Research Council (Grant Number 92091). Support was also provided by the National Institute for Health Research through the Primary Care Research Network.
